# The Prohibition of Suicide and Its Theological Rationale in Catholic Moral and Canonical Tradition: Origins and Development

**DOI:** 10.1007/s10943-023-01900-w

**Published:** 2023-08-29

**Authors:** Stanisław Adamiak, Jan Dohnalik

**Affiliations:** 1 Faculty of Theology, Nicolaus Copernicus University, Pl. Bł. Frelichowskiego 1, 87-100 Toruń, Poland; 2 Faculty of Canon Law, Cardinal Stefan Wyszyński University, Warsaw, Poland

**Keywords:** Suicide, Catholic moral theology, Catholic ethics, Canon law, Prohibition of funerals

## Abstract

This paper analyzes the nuances of the Roman Catholic Church’s opposition to suicide. First, we analyze biblical suicide cases, showing that these were not usually met with clear condemnation. Next, we show the development of the Roman Catholic Church’s moral teachings, with special attention to Augustine. The canon law of the Middle Ages still made some distinctions regarding motivation, but at this point, Roman Catholic funerals started to be refused to those having committed suicide as discouragement to others. This was maintained by the Council of Trent. In the twentieth century, the Roman Catholic Church followed modern psychiatry in attributing suicide to mental illness, and the ban on Roman Catholic funerals was lifted. Currently, the Roman Catholic Church tries to discourage suicide while accompanying people in need.

## Introduction

The opposition of the Roman Catholic Church to suicide is well known, and its long-standing ban on funeral rites for suicides has been the most visible sign of it for a long time. The topic of suicide prevention and the role of religion for this purpose is extremely important. In this context, it seems crucial to show the historical, theological and legal sources of the current teaching of the Catholic Church, which unequivocally opposes suicide. At the same time, there are many oversimplifications in the colloquial understanding of this topic regarding the history of the Church’s doctrine, law and practice on the subject. Therefore, a thorough tracing of the evolution of the teaching and the norms of the canon law on the subject, in dialogue with the social and psychological knowledge of a given historical period, will allow for a better understanding of the Catholic Church’s position today, which affects the conduct of a billion of its faithful. Even if social research does not show a clear and decisive influence of moral doctrine alone on the decision to commit suicide, a deep conviction of its moral evil is an important component of religion’s contribution to suicide prevention (Potter, 2021).

Therefore, in this study, we will show the ambiguous biblical mentions of suicide. This will be followed by an exposition of the teaching of Augustine of Hippo, whose role in unequivocally emphasizing the moral evil of suicide as self-murder was crucial. After that, the teaching of medieval canonists will be presented. They tried to combat suicide both through the practice of sacramental confession, which was supposed to be a cure for despair, and by strong sanctions against those who committed the act, such as the prohibition of Catholic burial. In modern times, the Church has upheld its doctrine and legal norms, while paying increasing attention to individual circumstances that justify departures from the strict letter of the canon law. Today, this line is continued: While the Church’s moral teaching continues to show the unequivocal evil of the act, developments in psychological and social sciences have resulted in a lenient approach to the personal responsibility of perpetrators and led to the abandonment of strict prohibitions on their burial. Toward the end, the authors attempt to confront historical testimonies and social research that indicate the possible impact of the moral teaching of the Catholic Church on suicide prevention.

## Method

This article first uses the historical–critical method, which consists of a thorough analysis of biblical, patristic, canonical and liturgical documents in their original context to determine the prevailing teaching and practice of the Roman Catholic Church during different periods of history (Rybińska, 2015). We also use inductive reasoning to draw conclusions from the texts we analyze. This method was also applied to contemporary documents to determine the current teaching of the Catholic Church in terms of moral theology and norms of canon law (Gałkowski, 2011). This reasoning was complemented in the final chapters by the comparative method, which consisted of confronting the above-mentioned findings with the conclusions of contemporary social, psychological and theological research, to discuss the possible correlation between religiosity and suicide and the effectiveness of the Church’s efforts to prevent suicide (Freiberger, 2019).

## Biblical Background

Although treating suicide as a grave sin often seems obvious, it is less clear when we take the Bible as the only source of moral teaching. Apart from the general prohibition on murdering humans, there is no explicit condemnation of suicide as such in the Bible. Thus, lacking such an imperative or prohibition, we turn to the moral role of the Biblical narratives. There are altogether less than ten unambiguous examples of suicide in the Bible: six in the Hebrew Bible, from one to three in the Apocrypha (accepted as canonical by the Roman Catholic Church and not by the Reformed communities), and one in the New Testament (Koch, 2005). Actual cases of suicide are rarely described, and when they are described, they are not met with outward condemnation (Barraclough, 1992).

Some of the Old Testament’s suicides are not very well known. King Abimelech, mortally wounded by a millstone during the siege of Thebez, ordered his armor-bearer to dispatch him to avoid the suggestion that he had been slain by the woman who had thrown the stone (King James Bible, 2017, Judges 9:52–54). The prophet Ahithophel hanged himself after betraying David (2 Samuel 17:23). King Zimri burned his house down around himself after a military defeat (1Kings 16:18). Then, the more familiar stories come, about Saul and his armor-bearer who both killed themselves after the lost battle against the Philistines (1 Samuel 31:4–6; 1 Chronicles 10:1–6) and about Samson who killed himself while taking down with him scores of Philistines (Judges 16:28). Finally, there is, of course, Jesus’s disciple Judas, although it is only in Matthew’s Gospel that he kills himself (Matthew 27:3–5; cf. Acts 1:18). Apart from the situations listed above, John 8:22 can also be treated as proof that the idea of suicide, imputed here to Jesus by a group of Jews, was looked at by the Jews with horror. On the other hand, the mass suicide of the defenders of Masada in 73 CE and some stories from the Talmud show that in certain circumstances, especially when faced with imminent death or rape at the hands of an enemy, suicide was deemed an acceptable option by Jews of the first and second centuries CE (Kaplan, 2021).

As far as the Apocrypha are concerned, there is the story of Eleazar, who died while killing a battle elephant during a battle against Seleucid forces (1 Maccabees 6:46); of Ptolemy Macron, a Greek general who tried to treat the Jews kindly was therefore accused in front of his king, and as a result poisoned himself (2 Maccabees 10:13); and of the Jewish hero Rasis, who was a respected citizen of Jerusalem, who opposed five hundred Greek soldiers sent to arrest him, and who threw himself from a height when he was surrounded, later literally eviscerating himself lest he be apprehended by his enemies (2 Maccabees 14:37–46).

Judas and the majority of the people from the Old Testament mentioned above were not examples to be followed for many reasons, with suicide only crowning their sins. Judas and Ahithophel were traitors. Abimelech’s cruelty and craving for power were clearly condemned. Zimri was in fact one of the short-lived usurpers in the brutal sequence of coups in the Kingdom of Israel. However, the cases of Samson and Saul are more problematic. Both were heroes chosen by God who later failed and fell from His favor. However, taking their own lives is not presented as one of their major faults. Saul’s death could be justified by his unwillingness to fall into the hands of his enemies, and Samson took not only his own life but also the lives of numerous enemies. Further, neither moral approval nor disapproval is connected to the suicide of Saul’s squire, which was undertaken mainly as an act of solidarity with his master.

The examples from the Books of Maccabees are the most problematic. First, it is difficult to say whether to treat the death of Eleazar as a suicide. There are no such doubts regarding the death of Ptolemy Macron, but this death is not met with condemnation. Then, in the case of Razis, his behavior is met not with condemnation but approval, as shown by the fact that, as he died, he expressed his hope in resurrection and future life.

## Augustine’s Equation of Suicide with Murder

Before the fourth century CE, the moral status of suicide did not have a central place in Christian thought, and when it was considered, it was not always met with outward condemnation (Amundsen, 1989). Lactantius, a Christian author from the beginning of the fourth century, was probably the first to explicitly link suicide with the commandment “Thou shalt not kill.” However, it was Augustine (354–431 CE), one of the greatest Fathers of the Western Church, who presented Catholic Christianity with a systematic reflection on suicide and its total condemnation under any circumstances. To do so, he tackled several possible counterexamples; the topic appeared in Augustine’s writings several times, but the most systematic exploration came in the first book of the “City of God” (Bels, 1975). Of course, Augustine was not discussing suicide undertaken out of cowardice, laziness, despair, contempt for life, or any other base motives (Chabi, 2020). What he dealt with was the concept of a “noble suicide,” as presented in classical literature (Ortiz, 2019).

In the “City of God,” Augustine refers to several examples of the famous suicides of Romans, especially Lucretia and Cato. However, he underlines that they cannot be followed by Christians. His reasoning probably had not only Christian but also Platonic ideas behind them: The will to exist is the only way for human beings to achieve true happiness, and the affirmation of suicide means considering a certain existence worthless (Minois, 2001; Sato, 2015). Augustine was also afraid that suicides could have “contagious effects” (Ortiz, 2019).

He also tackled Biblical examples. He extenuated Samson, because his action was not directed at his own death as its main purpose. However, Augustine added that not all of the actions of the saints were laudable. Augustine had to confront another problem: suicides whose actions were caused by their fidelity to God. This is at least partly the example of Samson, and even more of Razis. Was his death a suicide or a martyrdom? Where is the border between the two? This is among the questions that were hotly disputed by Christians amid the persecutions of the third and fourth centuries: When faced with the possibility of martyrdom, is it permissible for a Christian to try to avoid it, or, oppositely, should one actively seek it?

The official Church position was that it is allowed and even encouraged to flee persecutions and that it is morally wrong to provoke them. Although the majority of the examples of voluntary martyrdom come from the later literature and not from historical accounts (Cholewa, 2012), the declarative readiness for martyrdom was a feature that characterized the Donatists. In fourth-century North Africa, the Donatists constituted one of two Christian denominations; the Catholics, among them Augustine, constituted the second. The rivalry between the two Churches sometimes took the form of bloody conflict, with the Roman troops intervening at times on the side of the Catholics. The Donatists venerated the victims of such clashes as martyrs. However, the Catholics accused the Donatists of also venerating suicides who killed themselves in an imitation of martyrdom (Adamiak, 2019).

The perpetuation of this correlation between the Donatists (and especially their radical fringe, the Circumcellions) and suicide may have been one of the greatest successes of Catholic propaganda. A modern scholar, Shaw, writes that since at least 390 CE, suicide has been the “hard core” of schismatic resistance. In fact, if one were to take literally some of Augustine’s statements, one could imagine that the peaks of the cliffs of Africa were full of Donatists, or at least Circumcellions, all the time, going there en masse to throw themselves from the precipices and thereby gain the glory of martyrdom. The motif of throwing oneself from the precipices recurs again and again as, according to Augustine, a favorite form of this pious suicide committed by the Donatists. One can certainly see in this an echo of the events of the year 347 CE, when the deaths of two of the most famous Donatist martyrs, Donatus of Bagai and Marculus, undoubtedly had something to do with falling from a height. The Catholic Council of Carthage of 348 CE also forbade the worship of “those who throw themselves down” (Shaw, 2011, p. 740).

The problem, however, is that apart from the tirades of the Catholics, not a single event can be found and confirmed that could clearly be said to have actually constituted a conscious and voluntary suicide for the glory of martyrdom. Of course, everything depends on the exact definition of suicide, especially whether it includes death while fleeing from oppressors or voluntarily exposing oneself to persecution. Augustine undoubtedly thought of both as kinds of suicide, which is why he could so strongly condemn the Donatists as suicidal. Nevertheless, the only contemporary case he mentions is the story of the Donatist presbyter Donatus, who, fleeing from the Catholics, threw himself into a well and survived after being pulled out by his persecutors. As well, Augustine suggested that Marculus was a suicide since Roman law did not provide for executions by throwing oneself off of cliffs. On the other hand, Augustine writes that not only Catholic but also Donatist synods forbade the veneration of suicides as martyrs (Augustine, 401, v. 3, 54).

Undoubtedly, our picture of the Donatists’ willingness to take their own lives is influenced by the siege of their basilica at Timgad in 419 CE. At the time, Bishop Gaudentius threatened to set the basilica on fire and die with all those present there rather than surrender to the soldiers. It should be remembered that this story is known only through the accounts of Augustine, who himself learned the details secondhand. It is possible that Gaudentius uttered his threats convinced that the army was going to use force anyway. In any case, excavations at Timgad have not confirmed any great destruction from the early fifth century.

This last occasion of Gaudentius’s threat of mass suicide caused the letter by Augustine to Dulcitius, a Roman officer in charge of clearing the basilica. Augustine wrote to him saying that he had already explained many times why suicide is wrong but that indeed he had yet to respond to the argument from the Bible apparently employed by Gaudentius: the example of Razis. Therefore, he responded to it extensively in that letter. In doing so, he did not try to use allegorical exegesis, challenge the facts, or find other extenuating circumstances. No, Augustine said squarely and flatly that Razis did commit suicide and that whatever his motives, it was wrong and an example to avoid rather than follow:

In [books of the Old Testament,] there are many actions of those persons who are praised by the truth of those writings, but they are either not suitable to the present time or were not correctly done even at that time. Such is the act that this Razis committed upon himself. ... These actions are often praised in the books of the pagans. But though this man was praised in the Books of the Maccabees, his action was reported, not praised, and it was set before our eyes, as if it were, for judgment rather than for imitation. We should certainly not judge it by our own judgment, which we could also have as human beings, but by the judgment of sober teaching that is clear even in the old books. This Razis was certainly far removed from those words where we read: “Accept whatever is done to you, and endure it in sorrow, and have patience in your humiliation” (Ecclesiasticus 2:4). This man, then, was not wise in choosing death but impatient in bearing humiliation. ... These are great acts, but not good ones. After all, not everything that is great is good, since there are also great sins. (Augustine, 2004, *ep.* 204, pp. 6–8)

In this way, partly prompted by his ecclesiastical opponents, Augustine “closed” the teachings of the Catholic Church on suicide, not leaving any loopholes.

## Medieval Teachings

The reflections of Augustine were accepted by medieval authors. The first text we should consider here is the *Decretum Gratiani.* It was a collection of canonical and theological texts from the first millennium, compiled by Gratianus, an Italian monk, around 1140 CE. Augustine’s long text from “The City of God” was quoted by Gratian and preceded by his short sentence, “But no one is allowed to kill himself by any authority of the law” (Gratianus, 1959, col. 933). This snippet has been included between different texts about killing. It is very characteristic not only of Gratian but of all medieval sources that the word *suicide* was not used, but rather *killing oneself* (Schmitt, 1976). This was to emphasize the immoral aspect of suicide, which goes against the divine commandment “Thou shalt not kill.”

Augustine was the most important but not the only Father of the Church who tackled the issue of suicide. Leo the Great (400–461 CE) referred to Judas, showing that his sin was twofold: first by betraying Jesus and then by killing himself. The wrongness of the latter consisted in its being an act of desperation instead of penance (Leo the Great, 1846, col. 320; Maragno, 2016). It is not a coincidence that this text is included in a special fragment of the second part of Gratian’s decree, which is the treatise on penance. The juxtaposition of Judas’s suicide with the repentant Christian is a classic image of medieval devotional literature. In fact, these are two attitudes toward the sadness caused by sin, of which the attitude of the penitent is salutary, while that of the suicide is a desperate and evil way out. That is why medieval textbooks for confessors show confession as a place of counteracting sadness, anger and despair and thus also a place for preventing suicide (Schmitt, 1976). From this perspective, the adoption by the Fourth Lateran Council in 1215 CE of the law that required annual confession was also the Church’s response to spiritual causes of suicide (Dohnalik, 2015).

The other important aspect of the *Decretum Gratiani* was its insistence on the denial of funerary rites for suicides (Gratianus, 1959, col. 934). Gratianus quoted the rule that was expressed for the first time by the Council of Braga in 561 CE: “It was agreed that those who voluntarily bring death upon themselves either by iron or by poison or by the precipice or by hanging or in any other way, that no commemoration should be made for them in the offering, nor should their corpses be led to the burial with psalms” (Baron & Pietras, 2020, p. 17). The same was later confirmed by the Council of Auxerre in 578 CE and was repeated several times afterward, although there were also some regulations that allowed for the rites (Barry, 1997). The penitential books from the seventh and eighth centuries prohibited the saying of Mass for those who killed themselves by their own will, but Mass could be said if “they lost their mind because of a temptation or they killed themselves out of madness” (Baron & Pietras, 2011, p. 124). The long penance was prescribed for those who unsuccessfully attempted suicide (Baron & Pietras, 2011, p. 408). It seems that the main intention of the prohibition of ecclesiastical burial for suicides was not punishment of the dead but prevention of suicide.

The most important medieval theologian, Thomas Aquinas (1225–1274), upheld these ideas (McEvoy & Rosemann, 1993). Suicide was described by him as a grave sin against the Fifth Commandment and against the virtues of justice and charity (Masi, 1952). First, everyone has the natural duty to preserve his life and love it. Second, suicide is a crime against the human community, which is hurt by the death of one of its members. Third, it is a sin against God, who gave life to humans and is the only one allowed to take that life (Aquinas, 1947; Capello, 1958).

Even if Dante Alighieri in “The Divine Comedy” tried to acquit pagan suicides such as Cato or Lucretia, arguing in this way with Augustine (Schwebel, 2018), there were no exceptions in the new Christian reality. The sanction was obviously mainly in the supernatural realm as eternal condemnation, but there was also a more earthbound aspect of it: the prohibition of funeral rites. This was not the only earthbound sanction. Secular rulers added their own punishments for suicide, most importantly the forfeiture of the property of those who attempted or committed suicide. In this way, “the cooperation of Church and state created more effective impediments to suicide than had ever been seen in European history” (Barry, 1997, p. 536).

## The Refusal of Funerals as a Means to Prevent Suicides in Modern Times

After the shocks of the Reformation period, the second half of the sixteenth century brought a series of regulations reforming the life of the Roman Catholic Church. They followed the Council of Trent (1545–1563), which tried to impose strict rules on all areas of religious life. Among the liturgical reforms following the Council of Trent was the imposition of the unified “Roman Ritual” for the entire Church in 1614. It prescribed, among other rites, the rite of a Catholic funeral and included the list of people to whom a Catholic funeral should be denied: non-Catholics, public sinners, victims of duels, and suicides, if they had not given signs of penance prior to death. The exact wording addressed “those who killed themselves because of desperation or strong passion (*iracundia*), unless, as it happens, it resulted from insanity” (Wolczko, 2019, p. 14). It should be noted that both despair and anger here are probably understood in the medieval sense, as moral vices and sin-related diseases of the soul, and not as emotional and psychological forces (Schmitt, 1976). On the other hand, all disorders of a mental nature are included here in the word *insanity*.

However, the later commentators of this rule explained that although the previous deliberation clearly indicated that the prohibition on funeral rites for suicides should be applied, there may also be extenuating circumstances, such as fear, escaping from other evils, ignorance of the situation, and *furia*, which was considered a mental illness. The instructions of the Roman Inquisition were that in cases of doubt, a Catholic funeral should be granted, albeit without solemn ceremonies. It may be noted that the rules regarding victims of duels were to be applied much more rigorously (Chwastyk, 2010).

The prohibition of funeral ceremonies to suicides was maintained in the 1917 Code of Canon Law, the first modern codification of Church law. Canon 1240 of the Code gives the list of those to whom Catholic funerals should be denied unless signs of penance have been given before death. The third point of the first paragraph of the canon in question speaks of those who killed themselves *deliberato consilio.* It is no coincidence that, in the canon, there is no mention of despair or anger as situations in which a funeral should be refused, as is present in the 1614 ritual. Meanwhile, the wording *deliberato consilio* used in the canon meant a totally conscious and free choice, excluding not only mental illness in the clinical sense but also mental disorders and deep emotional problems that weaken discernment and freedom of decision. It is clear that at this point, the 1917 Code of Canon Law stands already in opposition to the stricter rules of the Ritual of 1614 as a result of a better understanding of psychological dynamics (Szwagrzyk, 1961).

Canonists emphasized that full accountability for such an act was needed to refuse a funeral. There was no question of such responsibility if the act was committed under the influence of fear or any other strong emotional state that prevented calm reflection. The opinion of theologians and canon lawyers of the time went in the same direction: All circumstances that could have led to the act of suicide not being totally deliberate should be considered, and it was better in ambiguous cases that the funeral be granted rather than denied (Gennaro, 1931; Szwagrzyk, 1961).

## The Development of Psychology and the Current Attitude of the Catholic Church to Suicides

As shown in the previous paragraph, the Catholic Church was not blind to the variety of human motivations behind suicidal acts. The Church’s understanding deepened with the development of psychological and psychiatric sciences (Dine, 2020). After the Second Vatican Council (1962–1965), several aspects of Catholic teaching and practice were revised. The new rite of funerals was published in 1978. It did not mention suicide at all. However, instructions and explanations from local bishops’ conferences followed. For example, the instruction of the Conference of the Bishops of Poland from May 5, 1978, explained in point 13, “According to the general opinion of psychiatrists, suicides are not fully responsible for their act. Therefore, they are not denied a Catholic funeral if during their lives they have shown devotion to the faith and the Church. The situation should be explained to the participants of the funeral. If there is serious doubt, the Ordinary should be consulted. A suicidal person who caused a scandal before attempting his own life should be treated as an open sinner” (Polish Bishops’ Conference, 1978; cf. Kołodziej, 2019).

Canon 1184 of the new (and still in place) 1983 Code of Canon Law has reduced the group of people to whom a Catholic funeral should be denied (Wolczko, 2019, p. 17–20). Suicides were not included among them. The only time when suicide is mentioned in the current Code of Canon Law is the Canon 1041 § 5, when it is considered one of the irregularities for receiving orders (Jaroszyński, 2011).

Bearing in mind the common opinion of psychiatrists, the principle is changed here compared to the previous, 1917 Code of Canon Law. Currently, suicides are not denied a Catholic funeral as a rule, provided that they are entitled to it under general rules. Some authors argue that deliberate suicides should still be denied burial, as they fall under the category of public sinners (Dyduch, 2017, p. 94). However, one should bear in mind the change to the funeral liturgy itself, which is now a request for God's mercy for the deceased rather than an honoring of Him through the rite, as well as the evolution of the social approach to the funeral liturgy (Power, 1985). It seems that in the current situation, Barry (1997, pp. 549–550) is right in saying that more good for suicide prevention can come from allowing church funerals while clearly teaching the inadmissibility of the act.

The Catechism of the Catholic Church was published in 1992 (final version in 1997), and it upholds the traditional moral teaching:

Suicide contradicts the natural inclination of the human being to preserve and perpetuate his life. It is gravely contrary to the just love of self. It likewise offends love of neighbor because it unjustly breaks the ties of solidarity with family, nation, and other human societies to which we continue to have obligations. Suicide is contrary to love for the living God.

If suicide is committed with the intention of setting an example, especially to the young, it also takes on the gravity of scandal. (Catechism of the Catholic Church, 1997, no. 2281–2282).

This strong moral affirmation is followed by the assessments of modern psychiatry in attributing suicidal tendencies to mental disorders: “Grave psychological disturbances, anguish, or grave fear of hardship, suffering, or torture can diminish the responsibility of the one committing suicide” (Catechism of the Catholic Church, 1997, no. 2282).

Thus, we may summarize the current position of the Roman Catholic Church in this way: Suicide is mostly seen as resulting from psychological difficulties, which strongly limits moral accountability. However, if a suicide was consciously conceived as something good, for example as a great, noble act, then the teaching of Augustine that condemns it remains valid. An important manual of Catholic ethics at the turn of the twenty-first century writes that suicide in itself is always morally wrong, regardless of intention, be it despair or heroic goals (Ślipko, 2021).

## The Efficacy of the Church’s Role in the Prevention of Suicide

Many scholars are convinced that, in the past, the teachings of the Catholic Church and its practices have had a strong effect on the prevention of suicide. Shaw (2011, p. 724) states, “There is little doubt that the impact of Catholic Christian and Islamic ideology has had a long-term depressing effect on self-killing in populations that subscribe to their value systems.”

The moral teachings of the Church have always been presented mainly by the clergy. Two categories of clergy, in particular, have been faced with moral dilemmas regarding suicide: military and hospital chaplains. The immorality of suicide under any circumstances was preached by Catholic military chaplains in Poland before the Second World War, e.g., by the military bishop Józef Gawlina, who underlined that suicide was against one’s duties toward God, the fatherland, family, and the love of oneself (Dohnalik, 2017). Such teachings directly influenced the decision of the Polish resistance commanders not to order their fighters to commit suicide when captured by Nazi Germans. The decision was hotly debated because it puts at risk the integrity of military secrets, but the opinion of the chaplains that no one can oblige another to commit suicide, even to keep the most important secrets, prevailed. Even if some members of the resistance, faced with the threat of torture and the danger of betrayal, decided to commit suicide (Cholewa, 2012), many were saved by the conviction that people of character could endure severe suffering with God's help, and that suicide is an unacceptable escape from such suffering (Woroniecki, 1986, pp. 173–174). A hero of the anti-German resistance, Jan Nowak Jeziorański, wrote later in his memoirs that many people imprisoned and tortured by Nazi Germans preserved their lives due to the deep conviction from family upbringing that one is not allowed to take one’s own life (Nowak Jeziorański, 1993, p. 167).

As well, the case of Lithuania is interesting here. Before the Second World War, along with Catholic Poland, Lithuania had much lower suicide rates than its Protestant neighbors Latvia and Estonia. However, today Lithuania is among the leading countries in terms of the prevalence of suicide. Gailiené claims that two factors seem to be most important: the heroicization of the suicides of resistance fighters and the experience of the long-term politics of atheization (Gailiené, 2018).

Contemporary analyses show that religion is a mitigating factor toward the number of suicides (Sisask et al., 2010). Interestingly, deeper involvement in church communities decreases suicide risk for Catholics but increases it for Protestants (Moksony & Hegedűs, 2019). The Republic of Ireland, a strongly Catholic society, has experienced a significant increase in suicide while religious practice has declined (Kelleher, 1999). A study from the USA distinguishes between the content of religious beliefs, religious affiliation and participation in practices. Not all the results are conclusive, the authors emphasize the variation of different faiths and the need for more in-depth research, but they confirm that religious practices have a positive impact on the prevalence of suicide attempts and can reduce suicides (Lawrence et al., 2016). Another study conducted recently in three regions of Austria and Italy tried to consider different dimensions of religiosity and spirituality. According to the researchers, so-called spiritual well-being is the most important component of spirituality that prevents suicide, while regional differences in suicide rates require further research (Stefa-Missagli et al., 2020). Also, a recent study from the Philippines highlights the correlation between spiritual well-being and effective suicide prevention (Gozum & Guttierez, 2023).

Active participation in religious life is negatively correlated with the probability of suicidal behavior in Poland (Kielan et al., 2017). One Polish author emphasizes the importance of correct anthropology and life affirmation for suicide prevention (Wolski, 2019). On the other side, the Catholic clergy in Poland do not feel adequately prepared to fulfill this role (Czabański, 2017). In the Philippines, after the difficult experience of the rise of suicide numbers during the COVID-19 pandemic in this country there is the Church project of preparing specialized teams for its prevention (Gozum & Guttierez, 2023).

## Limitations of the Study

This paper tried to analyze the evolution of the view of suicide by the Catholic Church. It is very difficult to assess the efficacy of the Church’s discouragement of suicide in the past ages, given the lack of any precise data. Even today, the correlation and relationship between religiosity and suicide has only been studied in some countries. More research would certainly allow more confident conclusions to be reached. On the other hand, religiosity is only one of the factors that may influence regional differences in suicide tendencies, so any conclusions should be approached with due caution. Then, we have omitted the issue of assisted suicide altogether in this paper. The Catholic Church’s teaching generally paragons it with suicide or murder, but of course the practice of euthanasia raises several moral, religious and bioethical issues (John Paul II, 1995, pp. 65–66).

## Conclusion

Over the centuries, the Church has tried to counter suicide in three ways: through ethical teaching, through legal sanctions and by bringing people out of despair, especially through sacramental confession. In this study, we have shown the patristic roots of the Church’s moral teaching on suicide. The logic of the Augustinian understanding of suicide as the unacceptable killing of oneself has remained unchanged ever since. Although the historical and sociological studies cited are not conclusive, they nevertheless show the unequivocal teaching on the matter as an important factor in reducing suicide in the Catholic environment.

We have shown the evolution of the norms of the canon law of the Catholic Church. We were able to see that they remained in dialogue with the contemporary understanding of human nature. Nowadays, thanks to the development of psychological and psychiatric sciences, we better understand that premeditated and prepared suicide is rare, and the Church almost no longer applies legal sanctions related to the refusal of burial.

The final element of suicide prevention is accompanying people in states of emotional, life and mental difficulties. It still seems relevant to see the great power to counter despair and hopelessness in a wisely administered sacramental confession, where the human and spiritual dimensions meet. The Church, which according to Pope Francis is supposed to be a “field hospital” (Cavanaugh, 2016), must not limit itself to ethical messages, let alone criminal sanctions, but should help psychologists and psychiatrists heal the spiritual wounds and deficiencies that can drive people to suicide. This can be done not only by pastors but also by all the faithful, as Durkheim has already noted in his classic study of suicide: It is not so much doctrine as the cohesion of the religious community that plays a role in its avoidance (Durkheim, 1897).
